# Association between exercise load, resting heart rate, and maximum heart rate and risk of future ST-segment elevation myocardial infarction (STEMI)

**DOI:** 10.1136/openhrt-2023-002307

**Published:** 2023-07-17

**Authors:** Edvin Helleryd, Araz Rawshani, Aidin Rawshani, Nellie Hjärtstam, Anna Myredal, Kristofer Skoglund

**Affiliations:** 1 Department of Molecular and Clinical Medicine, University of Gothenburg, Goteborg, Sweden; 2 Department of Cardiology, Sahlgrenska University Hospital, Goteborg, Sweden

**Keywords:** Acute Coronary Syndrome, Myocardial Infarction, Chest Pain

## Abstract

**Objective:**

This study aimed to examine the association between exercise workload, resting heart rate (RHR), maximum heart rate and the risk of developing ST-segment elevation myocardial infarction (STEMI).

**Methods:**

The study included all participants from the UK Biobank who had undergone submaximal exercise stress testing. Patients with a history of STEMI were excluded. The allowed exercise load for each participant was calculated based on clinical characteristics and risk categories. We studied the participants who exercised to reach 50% or 35% of their expected maximum exercise tolerance. STEMI was adjudicated by the UK Biobank. We used Cox regression analysis to study how exercise tolerance and RHR were related to the risk of STEMI.

**Results:**

A total of 66 949 participants were studied, of whom 274 developed STEMI during a median follow-up of 7.7 years. After adjusting for age, sex, blood pressure, smoking, forced vital capacity, forced expiratory volume in 1 s, peak expiratory flow and diabetes, we noted a significant association between RHR and the risk of STEMI (p=0.015). The HR for STEMI in the highest RHR quartile (>90 beats/min) compared with that in the lowest quartile was 2.92 (95% CI 1.26 to 6.77). Neither the maximum achieved exercise load nor the ratio of the maximum heart rate to the maximum load was significantly associated with the risk of STEMI. However, a non-significant but stepwise inverse association was noted between the maximum load and the risk of STEMI.

**Conclusion:**

RHR is an independent predictor of future STEMI. An RHR of >90 beats/min is associated with an almost threefold increase in the risk of STEMI.

**WHAT IS ALREADY KNOWN ON THIS TOPIC**

- Exercise capacity is inversely associated with the risk of death and acute myocardial infarction (AMI). No studies has examined the association between resting heart rate (RHR) or exercise capacity and future risk of ST elevation AMI, which reflects complete epicardial occlusions.

**WHAT THIS STUDY ADDS**

- This study shows that RHR is an independent predictor of future ST-segment elevation myocardial infarction (STEMI). A RHR of >90 beats/min is associated with an almost threefold increase in the risk of STEMI. Maximum achieved exercise load and ratio of the maximum heart rate to the maximum load were not associated with the risk of STEMI, which may be explained by the low load used in the UK Biobank.

**HOW THIS STUDY MIGHT AFFECT RESEARCH, PRACTICE OR POLICY**

- RHR, a readily available risk factor, is an important predictor of STEMI risk. The risk conferred by high RHR is comparable to conventional risk factors for AMI.

## Introduction

Coronary artery disease (CAD) is the leading cause of death and mortality worldwide. The prevalence of CAD in the Western population is approximately 15%.^1^ Acute myocardial infarction (AMI) generally results from ruptured, ulcerated or fissured atherosclerotic plaque.^2 3^ A range of factors modify the stability of atherosclerotic plaques, plaque progression, cap thickness, and balance between prothrombotic and antithrombotic factors. Plaque ruptures are substantially more common than acute coronary syndromes, highlighting the fundamental importance of prothrombotic and antithrombotic factors.^4^ Numerous studies have demonstrated that exercise level is inversely associated with the risk of AMI. Notably, a very strong inverse association between physical activity during leisure time, maximal oxygen uptake and the risk of AMI was reported by Lakka *et al*^5^; individuals in the highest tertile of exercise capacity had a 64% lower risk of AMI after accounting for a range of confounders. However, these studies are frequently limited by the lack of quantitative data on exercise levels, limited power and inability to distinguish between different acute coronary syndromes. Moreover, residual confounding is a complicated matter concerning exercise, meaning that it is difficult to isolate the intrinsic effect of cardiorespiratory exercise because it may be accompanied by a host of other health-promoting behaviours.

AMI comprises a broad spectrum of coronary events, including myocardial infarction in non-obstructive coronary arteries (eg, secondary to anaemia), spasm-induced infarctions, demand–supply imbalance in obstructive or non-obstructive arteries, and plaque ruptures with partial or total occlusion. ST-segment elevation myocardial infarction (STEMI), caused by complete epicardial occlusion in most cases, is possibly the most clearly defined acute coronary syndrome.

Resting heart rate (RHR) is associated with all-cause and cardiovascular mortality.^6 7^ This effect was found to be more pronounced in men than in women.^7^ Current models predict cardiovascular events and overall mortality using the submaximal exercise test in the UK Biobank^8–10^; however, there is no clinical prediction that predicts STEMI using the submaximal exercise test. We used the UK Biobank to study the association of the achieved exercise workload and RHR with the risk of developing STEMI.

## Methods

### UK Biobank

The UK Biobank is an ongoing research project aimed at determining the causes of a wide spectrum of diseases. The database consisted of approximately 500 000 participants aged 40–69 years at inclusion. Recruitment was handled by 22 assessment centres. These centres recruited a nearby population through mail and local appearances. The UK Biobank database was enriched with outcome data by merging it with population-based registers from the National Health Service, which allows for the rapid retrieval of individual-level health-related data. The recruited individuals were examined at a local assessment centre for baseline assessment, which involved a digital questionnaire, an interview, physical measurements and biological sample collection.^11^ Biological samples were analysed and archived for future analyses.

### Data sharing statement

UK Biobank is accessible to researchers worldwide. Data access can be requested at UK Biobank - UK Biobank.

### Exercise testing

A submaximal exercise test was performed on 77 928 participants in a UK Biobank. These participants were the last to be recruited for the UK Biobank Study. The subset was made due to budget constraints, and the reasoning for selecting the last participants was due to the decision to include additional tests being made late into recruitment.^11^ The participants were stratified into risk categories, depending on previous cardiovascular conditions, chest pain at rest or during physical activity, blood pressure, weight, RHR, pregnancy, ability to walk and whether the participant had a pacemaker. There were five risk categories, numbered 1–5, where one was minimal risk and five implied that the submaximal exercise test must be avoided. The participants in categories 1–3 performed the exercise tests. Category 4 included only resting measurements. The absolute maximum workload was predicted for each participant based on age, sex, height, weight and RHR. The participants in category 1 and category 2 achieved 50% and 35% of their predicted absolute maximum workload, respectively. A lower and more constant workload was selected for category 3. For more information on risk stratification and test protocols, please refer to the cardiac assessment protocol of the UK Biobank.^12^

The submaximal exercise test consisted of three phases: the initial phase was a 15 s resting phase; the second phase started at a constant load (30 W for female participants and 40 W for male participants) and continued with a gradual increase in workload for 4 min until the goal load was achieved, and the final phase was a 1 min recovery phase. The submaximal exercise test was terminated early if the heart rate reached 75% of the age-predicted maximum heart rate or if chest pain, dizziness or presyncope occurred. The exercise phase could also be terminated if participants perceived muscle or joint pain or desired to end the test.

### Participant selection

We included participants who underwent a submaximal exercise test during their first assessment visit to the UK Biobank. Individuals with a history of STEMI, according to algorithmically defined outcomes for STEMI in the UK Biobank, were excluded. To study the maximum load and the ratio of maximum heart rate to maximum load, only risk groups 1 and 2 were used (ie, the groups that performed the submaximal exercise test). RHR was available for all participants who performed the submaximal exercise test.

### STEMI outcome

The occurrence of STEMI was retrieved using algorithmically defined outcomes from the UK Biobank, see supplent file 1. These studies used a combination of self-reported, hospital admission and death registry data. The International Classification of Diseases, 10th Revision, codes used for this process were I21.0, I21.1, I21.2, I21.3 I22.0, I22.1 and I22.8. The outcome has been validated, and the positive predictive value for STEMI is 71%–100%.^13^ In this study, we excluded patients with previous STEMI. The outcome was defined as survival with a timespan from the submaximal exercise test to STEMI, death, loss to follow-up or withdrawal of consent, whichever occurred first.

### Analysed variables and covariates

The maximum load, RHR and maximum heart rate were the main predictors of interest. The following covariates were considered: age, sex, systolic and diastolic blood pressures, weight, body mass index (BMI), spirometry values (forced vital capacity (FVC), forced expiratory volume in 1 s (FEV_1_) and PEF) and smoking status. The rationale of the chosen variables was to select biometric variables that were readily available to clinicians.

### Statistical analysis

Baseline characteristics are presented as mean values with SDs.

Three separate analyses were conducted for each predictor of interest, resulting in nine models. The first analysis was a Cox proportional hazards regression model, in which one of the examined variables was analysed and adjusted for covariates. The second analysis was also a Cox proportional hazard regression model with the predictor of main interest modelled using restricted cubic splines; this model was also adjusted for the covariates. In the third analysis, the predictors of men’s interest were categorised into quartiles and analysed using the same Cox model.

## Results

### Baseline characteristics

A total of 77 505 participants were referred for exercise testing, and 274 developed STEMI during the follow-up period. The median follow-up was 7.7 years. The mean age of the cohort was 56.8 (SD 8.1 years) at the start of follow-up, and 45.5% of the participants were male. Seventy-five per cent of the participants were placed in the lowest-risk category, which included exercise at 50% of the predicted tolerance, whereas 10.7% of the participants were placed in the fourth category, which only recorded the rest of the measurements. The mean maximum load was 72 W, and the RHR was 69 beats/min. The mean maximum heart rate was 110 beats/min (table 1). In the subgroup analysis of maximum load and the ratio of maximum heart rate to load, there were 66.949 participants from categories 1 and 2. Diabetes was prevalent in 5.6% of the participants. The mean BMI was 27.4 kg/m^2^.

**Table 1:** Baseline characteristics of the cohort

	Overall
n	77 505
Age (years), mean (SD)	56.83 (8.11)
Sex, male (%)	35 091 (45.3)
Max load, mean (SD)	71.71 (35.64)
Duration, mean (SD)	27.39 (33.97)
Resting heart rate, mean (SD)	69.28 (11.93)
Max heart rate, mean (SD))	110.35 (20.10)
Risk category (%)	
No category, ECG not to be done	13 (0.0)
Category 1, cycle rising to 50% level	58 156 (75.0)
Category 2, cycle rising to 35% level	8793 (11.3)
Category 3, cycle at constant level	2274 (2.9)
Category 4, at-rest measurement	8269 (10.7)
Systolic blood pressure, mean (SD)	139.96 (19.61)
Diastolic blood pressure, mean (SD)	81.96 (10.64)
Diabetes (%)	4293 (5.6)
Weight, mean (SD)	78.10 (16.04)
Body mass index, mean (SD)	27.39 (4.78)
FVC, mean (SD)	3.65 (0.99)
FEV_1_, mean (SD)	2.78 (0.78)
PEF, mean (SD)	388.39 (135.46)
Pack-years of smoking, mean (SD))	6.26 (13.64)
Alcohol frequency	
Prefer not to answer	87 (0.1)
Daily or almost daily	15 773 (20.4)
Three or four times a week	17 311 (22.4)
Once or twice a week	19 181 (24.8)
One to three times a month	8728 (11.3)
Special occasions only	9690 (12.5)
Never	6529 (8.4)

For numeric variables, mean value and SD are presented, and for discrete variables, the total number of participants for each variable is presented along with the percentage of total participants.

FEV_1_, forced expiratory volume in 1 s; FVC, forced vital capacity; max, maximum.

#### Univariate associations

In the univariate analyses, age (p <0.001), sex (p <0.001), duration of exercise test (p<0.001), maximum heart rate (p=0.002) and RHR (p=0.001) were associated with the development of STEMI (table 2). However, the maximum load was not significantly associated with STEMI development. Those who later developed STEMI had higher RHRs (72 beats/min vs 69 beats/min) but lower maximum heart rates during stress testing (106 beats/min vs 110 beats/min).

**Table 2:** Univariate analysis of all variables

	No STEMI	STEMI	P value
n	77 231	274	
Age (years), mean (SD)	56.82 (8.12)	59.98 (6.68)	<0.001
Sex, male (%)	34 880 (45.2)	211 (77.0)	<0.001
Max load, mean (SD)	71.73 (35.62)	67.77 (40.67)	0.067
Duration, mean (SD)	27.36 (33.93)	35.97 (42.21)	<0.001
Resting heart rate, mean (SD)	69.27 (11.92)	71.67 (12.91)	0.001
Max heart rate, mean (SD)	110.36 (20.10)	106.68 (20.57)	0.002
Risk category (%)			<0.001
No category, ECG not to be done	13 (0.0)	0 (0.0)	
Category 1, cycle rising to 50% level	57 986 (75.1)	170 (62.0)	
Category 2, cycle rising to 35% level	8745 (11.3)	48 (17.5)	
Category 3, cycle at constant level	2265 (2.9)	9 (3.3)	
Category 4, at-rest measurement	8222 (10.6)	47 (17.2)	
Systolic blood pressure, mean (SD)	139.93 (19.61)	146.97 (20.02)	<0.001
Diastolic blood pressure, mean (SD)	81.95 (10.64)	84.63 (10.71)	<0.001
Diabetes (%)	4260 (5.5)	33 (12.1)	<0.001
Weight, mean (SD)	78.08 (16.04)	82.52 (15.68)	<0.001
Body mass index, mean (SD))	27.39 (4.78)	28.00 (4.16)	0.036
FVC, mean (SD)	3.65 (0.99)	3.72 (0.90)	0.261
FEV_1_, mean (SD)	2.78 (0.78)	2.81 (0.73)	0.538
PEF, mean (SD)	388.31 (135.47)	413.02 (131.22)	0.005
Pack-years of smoking, mean (SD)	6.23 (13.61)	12.89 (19.07)	<0.001
Alcohol frequency			0.502
Prefer not to answer	87 (0.1)	0 (0.0)	
Daily or almost daily	15 717 (20.4)	56 (20.5)	
Three or four times a week	17 259 (22.4)	52 (19.0)	
Once or twice a week	19 120 (24.8)	61 (22.3)	
One to three times a month	8693 (11.3)	35 (12.8)	
Special occasions only	9649 (12.5)	41 (15.0)	
Never	6501 (8.4)	28 (10.3)	

Data are presented with the distribution of variables with future STEMI as the stratifying variable.

FEV_1_, forced expiratory volume in 1 s; FVC, forced vital capacity; max, maximum; STEMI, ST-segment elevation myocardial infarction.

### Multivariable analyses

#### Resting heart rate

**Figure 1:**
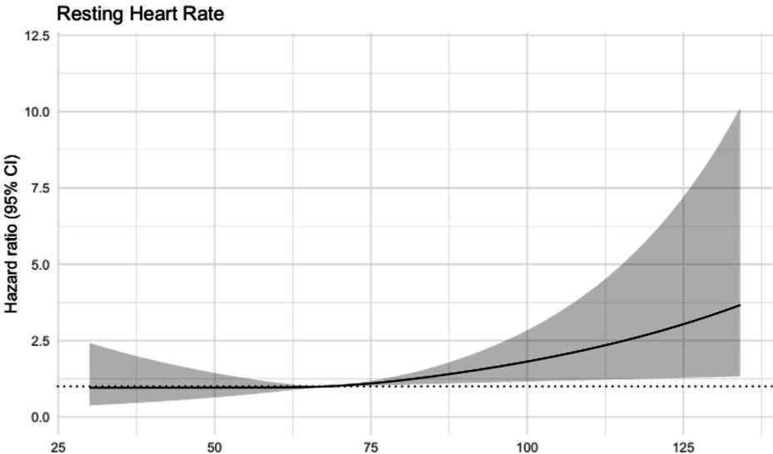
Cubic spline plot with three knots for the HR of RHR. The HR is presented on the Y-axis and RHR on the X-axis. The black line represents the HR for RHR. The grey area represents the 95% CI, and the dotted line represents 1 hour. RHR, resting heart rate.

**Figure 2:**
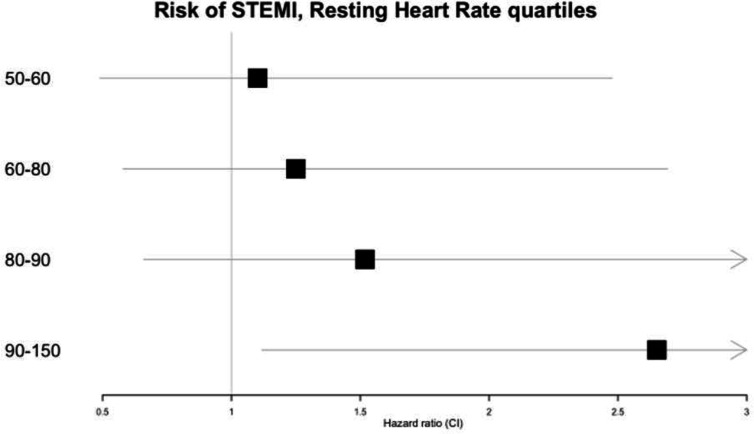
Forest plot of the HR for the five groups of resting heart rate (<50, 50–60, 60–80, 80–90, >90). The group <50 represents HR of 1 (ie, reference group). STEMI, ST-segment elevation myocardial infarction.

In the Cox model, which was adjusted for age, sex, blood pressure, pack-years of smoking, BMI, weight, FVC, FEV_1_, PEF and diabetes, we noted a p value of 0.015 for RHR, with a gradual increase in the risk of STEMI with RHR (table 3). Modelling the RHR with a spline function, we noted an HR of roughly 2.0 at a RHR of 115 beats/min, compared with the median (67 beats/min, figure 1). Figure 2 shows that the highest quartile (heart rate >90) had an HR of 2.92 (95% CI 1.26 to 6.77).

**Table 3:** Multivariate analysis of RHR

Characteristic	HR	95% CI	P value
RHR	1.01	1.00 to 1.02	0.015
Age	1.04	1.02 to 1.06	<0.001
Sex			
Female	–	–	
Male	5.72	3.76 to 8.70	<0.001
Systolic blood pressure	1.01	1.00 to 1 02	0.2
Diastolic blood pressure	1.01	0.99 to 1 02	0.5
Pack-years of smoking	1.01	1.01 to 1.02	<0.001
Body mass index	1.00	0.93 to 1.08	>0.9
Weight	0.99	0.97 to 1 01	0.3
FVC	0.68	0.44 to 1.04	0.076
FEV_1_	1.11	0.63 to 1 95	0.7
PEF	1.00	1.00 to 1 00	0.3
Diabetes			
No	–		
Yes	1.44	0.94 to 2.23	0.1

Data are presented with HRs, 95% CIs, and p-values for each variable in the model.

FEV_1_, forced expiratory volume in 1 s; FVC, forced vital capacity; RHR, resting heart rate.

#### Maximum load and ratio of maximum heart rate to maximum load

Neither the maximum load nor the ratio of maximum heart rate to maximum load showed any significant association with the risk of STEMI (p=0.113 and p=0.167, respectively).

## Discussion

The main finding of this study is that the RHR is a strong and independent predictor of STEMI. Although this confirms previous knowledge regarding the role of RHR as an important predictor of cardiovascular outcomes, this study adds novelty by demonstrating how RHR relates to a specific vascular condition, namely, STEMI. This outcome distinguishes itself from the other outcomes because it is caused by the complete and acute occlusion of an epicardial coronary artery. These events occur only when prothrombogenic factors far outweigh prothrombolytic factors in the blood,^4^ demonstrating that RHR is strongly associated with the risk of STEMI, given that an RHR of >90 beats/min was associated with a threefold increased risk of STEMI.

The magnitude of this association should be compared with conventional risk factors, notably the INTERHEART risk factors.^14^ Hypertension, diabetes, smoking and cholesterol level were the strongest predictors of AMI in the INTERHEART study. The ORs for diabetes, smoking and hypertension in the INTERHEART study were on par with the HR noted for RHR of >90 beats/min in the current study. Indeed, we demonstrated that the RHR, a readily available risk factor, is an important predictor of STEMI risk. This should prompt clinicians to consider RHR when performing risk assessments in patients with cardiometabolic risk factors. The RHR is easily obtained and is often accompanied by other measurements (ECG, automatic blood pressure measurements, etc).

Although there is rigorous evidence of RHR as an independent predictor of cardiovascular disease (CVD) in both men and women, the use of RHR as a predictor is omitted from the SCORE2 model and only mentioned in passing in the 2021 European Society of Cardiology guideline for CVD prevention.^15^ The AHA guideline for the primary prevention of CVD also omits RHR.^16^ However, AHA, ACC as well as ESC recommend several evidence-based medications that lower RHR (eg, beta blockers). Our study suggests that such interventions may be beneficial beyond the traditional view of how beta blockers exert their cardioprotective effects.

It is important not to consider this association as evidence of causation. The association between the RHR and the risk of STEMI may be explained by confounders. We made efforts to adjust for important confounders and obtained the aforementioned HRs after accounting for conventional risk factors. However, this does not rule out residual confounding factors that may have biased the results. It can be argued that the ultimate method for elucidating whether heart rate lowers the risk of STEMI is through randomised clinical trials with HR-lowering drugs. However, such trials are likely not feasible because the absolute risk of STEMI is very low in a population not treated with cardioprotective drugs, making the trial costly. A more feasible method would be to adopt a Mendelian approach, study gene variants that lower heart rate and study how they relate to the risk of STEMI.

Analysis of workload and maximal heart rate did not yield any significant association with the risk of STEMI. One possible explanation is that the protocol for submaximal exercise tests in the UK Biobank limits the workload in all patients, particularly those at higher risk. This is partly in contrast to clinical practice, in which submaximal or maximal tests are usually performed, meaning that patients are subjected to substantially greater stress when performing stress tests in routine practice. In the UK Biobank, each participant was assigned a target workload that was 35%–50% of their predicted maximum, which was achieved by 92% of the participants. It follows that 92% of the participants theoretically have a higher maximum load than that reported in the UK Biobank, making those measurements less useful for studying the associations between workload and CVD risk. Nevertheless, because the number of participants in the UK Biobank is very large and the workload is a continuous variable, it is theoretically possible to detect even small effects across the range of workloads tested. The fact that we were unable to detect such associations does not rule out their existence.

It is also possible that the relatively small number of STEMI events (n=274) was an important reason for the lack of significant associations between exercise capacity and the risk of STEMI.

### Limitations

The main limitation of this study is the protocol used to conduct the submaximal exercise test. Another limitation is that the analysed RHR comes from the resting phase before the submaximal exercise test, which likely does not reflect the lowest RHR in individuals owing to stress and anticipation before the test. The age spectrum in the UK Biobank is also a potential limitation because it does not reflect the overall population. The mean follow-up was 7.7 years, and only 274 cases of STEMI were included. A longer follow-up period might have increased the power and improved the precision of the estimates. The group that performed the submaximal exercise test was also limited to approximately 15% of the entire cohort in the UK Biobank, owing to budget constraints.

We conclude that RHR is a strong and independent predictor of STEMI, comparable to the conventional risk factors of AMI.

10.1136/openhrt-2023-002307.supp1 Supplementary data

10.1136/openhrt-2023-002307.supp2 Supplementary data

## Data Availability

Data may be obtained from a third party and are not publicly available. UK Biobank is accessible to researchers worldwide. Data access can be requested at https://www.ukbiobank.ac.uk.
