# Supplementary material for: Association between exercise load, resting heart rate, and maximum heart rate and risk of future ST-segment elevation myocardial infarction (STEMI)
Source: Open Heart. 2023 Jul 17;10(2):e002307. doi: 10.1136/openhrt-2023-002307 (PMC10357634; doi:10.1136/openhrt-2023-002307)
Supplement: Supplementary data [file openhrt-2023-002307supp001.pdf]

UK Biobank

Algorithmically-defined outcomes

(ADOs)

Version 2.0

www.ukbiobank.ac.uk
January 2022

The content of this work and its documentation was originally prepared by
UK Biobank Outcome Adjudication Group (members are named in relevant sections).
This revised document has been produced by UK Biobank's Data Analyst and Scientific teams.

Contents

1. Changes made to the algorithmically-defined outcomes 3
2. Introduction 4
3. Data sources and process 5
4. General algorithm 7
5. Using the algorithmically derived health outcome data-fields 8
6. Key points to note 9
7. Health outcome code lists 10
   a) Asthma 10
   b) Chronic obstructive pulmonary disease (COPD) 11
   c) Dementia 12
   d) Myocardial infarction (MI) 16
   e) Motor neurone disease (MND) 19
   f) Parkinson’s disease 20
   f) Stroke 24

Acknowledgements 28

Helleryd E, *et al. Open Heart* 2024; 10:e002307. doi: 10.1136/openhrt-2023-002307

1. Changes made to the algorithmically-defined outcomes

The UK Biobank Outcome Adjudication Group, in conjunction with clinical experts, developed and validated algorithms based on lists of clinical codes to ascertain a range of health outcomes – referred to as 'algorithmically-defined outcomes' (ADOs) in the Data Showcase.

The original algorithms for the ADO fields in Showcase have now been revised. The new ADOs use a standardised approach to defining health outcomes, which identify the earliest recorded date of a given health outcome irrespective of source (i.e. self-report, hospital admission, death). Another aspect to the new ADOs is that, additional encodings have been assigned to the source of the earliest event, so that for all ADOs except end-stage renal disease, information is provided on whether a code from a hospital or death record is in the primary position (leading cause of admission, or underlying cause of death) or is a secondary (contributory) cause of illness or death. This additional information will enable researchers to be more discerning about their selection of cases depending on the specific research question.

This document describes the updated algorithms used for the following health outcomes: asthma, chronic obstructive pulmonary disease, dementia, myocardial infarction, motor neurone disease, Parkinson's disease and stroke. This document replaces the condition-specific PDF resources that were originally provided on Showcase in 2018 (Resources 461, 462, 1518, 1747, 4124, 4125 and 6365). The algorithm for end-stage renal disease has also been updated in a similar manner, but because it is more complex, the documentation has been kept as a separate resource (Resource 8319).

Helleryd E, *et al. Open Heart* 2024; 10:e002307. doi: 10.1136/openhrt-2023-002307

2. Introduction

The algorithmically-defined outcomes contain data on probable cases of selected health conditions, obtained through algorithmic combinations of coded information from UK Biobank's baseline assessment data collection (which included data from participants on their self-reported medical conditions, operations and medications), along with linked data from hospital admissions (diagnoses and procedures) and death registries. The purpose of these derived data-fields is to help researchers to include health-related outcomes in their analyses without having to select lists of diagnostic and/or procedural codes and combine the different data sources themselves. The selected health outcomes are based on algorithms developed by the UK Biobank Outcome Adjudication group and aim to classify disease outcomes with high positive predictive value (i.e. a high probability that people identified as having a health-related event have indeed experienced that event). Where possible, we provide the best available information on the estimated positive predictive value of each source or combination of sources so that researchers can use this information in their analyses.

3. Data sources and process

Currently, the algorithm uses data sources from:

- UKB baseline assessment data (self-reported verbal interview)
- Linked hospital-admission data (HES APC - England, SMR01 - Scotland, PEDW - Wales)
- Death register data

The data included in the algorithms are taken from the baseline assessment touchscreen questionnaire and/or nurse-led interview and from linked national health-related datasets. Data from primary care linkages is not currently included (as these data are not yet available for the full cohort) but will be incorporated into future versions of the algorithms. To identify individuals who are likely to have been diagnosed with a certain health outcome, disease-specific codes were extracted from the relevant datasets, which can be found separately in this document for each specific condition or group of related conditions. The algorithm produces derived data-fields related to a participant having been diagnosed with that health condition: the date of the event and the relevant data source. The table below gives some examples:

**ID**	**Any stroke**		**Subarachnoid haemorrhage**		**…**	**Any MI**		**…**
	**Date**	**Source**	**Date**	**Source**	**…**	**Date**	**Source**	**…**
007	01.01.2015	Self-report						
009						01.03.2015	Hospital primary	
…	…	…						

Date refers to the earliest date of the health event identified in any of the combined datasets; **Source** refers to the dataset in which the event was identified. **'Self-reported only' Source** refers to participants who indicated in the nurse-led interview at their baseline assessment that they had been diagnosed by a doctor with that health condition and who had no relevant hospital admission diagnosis or procedure code prior to their date of recruitment into the UK Biobank cohort. The **Date** for **'Self-reported only'** events refers to the date at which the participant stated they were first diagnosed when asked at the baseline assessment. '**Death primary**' or '**Death contributory**' **Source** refers to participants with a relevant code in the death registration records with no baseline

self-report of the event and no relevant hospital admission diagnosis or procedure code. The **Date** for '**Death primary**' or '**Death contributory**' events refers to the date of death. **'Hospital primary**' or '**Hospital secondary**' **Source** refers to participants with a relevant diagnosis or procedure code in the hospital admission data, and who did not self-report this event at baseline (i.e. they are not classified as **'Self-reported only'** (see above). The **Date** for **'Hospital primary**' or '**Hospital secondary**' events is the earliest date of a relevant event within the linked hospital admissions data.

Definitions & Abbreviations

ADO	Algorithmically-defined outcomes
COPD	Chronic obstructive pulmonary disease
EHR	Electronic Health Records
HES APC	Hospital Episode Statistics - Admitted Patient Care (England)
ICD 9	International Classification of Diseases, Version 9 (SMR only)
ICD 10	International Classification of Diseases, Version 10
MI	Myocardial infarction
MND	Motor neurone disease
PEDW	Patient Episode Database for Wales
Read codes	A coded thesaurus of clinical terms used in NHS primary care since 1985 with a final update in April 2018. The system is no longer in active use owing to the phased introduction of SNOMED CT.
SMR01	Scottish Morbidity Records – General / Acute Inpatient and Day Case Admissions (Scotland)

4. General algorithm

I. All ADOs (except for end-stage renal disease) follow the same algorithm.

II. Using a predefined code list for each health outcome, for each individual the algorithm takes the earliest recorded date and its related data source, based on the following sources:

   a. **Hospital admission records:** One (or more) of the pre-defined ICD (9 or 10) codes included in HES APC, SMR01 or PEDW linked records in the primary or any secondary position; or specified combination of these codes as defined for a given health outcome;

   b. **Self-report at nurse interview**: The participant has self-reported the condition at baseline[1] interview and given the date of onset.*

   c. **Death certificate records**: one of the pre-defined ICD-10 codes as listed in the 'underlying cause' or 'secondary cause' fields.

III. In contrast to the original ADOs, health outcomes (including disease subtypes where applicable) are treated independently. For example, a participant with a record of two stroke subtypes (e.g. subarachnoid haemorrhage and ischaemic stroke) will have a record in each of the stroke subtype ADOs (a record in the subarachnoid haemorrhage ADO and in the ischaemic stroke ADO), as well as the stroke ADO.

IV. Data-Coding 300 indicates whether a hospital or death source derives from a code listed in the primary position (i.e. leading cause of hospital episode, or underlying cause of death) or a secondary (contributory) position.

***Note on self-reported dates:** When participants enrolled in the UK Biobank study, they underwent a verbal interview with a research nurse, in which they could 'self-report' medical conditions. The self-report date is taken from the UK Biobank field 20008 ("Interpolated Year when non-cancer illness first diagnosed"). At the verbal interview, UKB nurses were instructed to record either a year or an age at which the diagnosis occurred. Where an age was provided, a best-fit fractional year was then calculated. These have been rounded to one decimal place, and as such should be regarded as a close proxy to the reported date. Some dates of onset are missing. Where this is the case, and the baseline interview was the first data source to record the health outcome, the date of onset will be set to 1/1/1900.

[1] Only self-reports at baseline have been included in the algorithms, as subsequent self-report data are not available for all the cohort and most events will likely be picked up through record linkage.

5. Using the algorithmically derived health outcome data-fields

The algorithms are designed to enable the selection of cases of disease for a range of different research questions. The prospective design of UK Biobank makes it particularly suitable for studies involving incident cases (i.e. those first diagnosed or detected with a condition after recruitment to the study), but the algorithms identify disease cases diagnosed both before and after recruitment to UK Biobank, which might be of relevance to those performing genetic analyses. Researchers are advised to merge the algorithmically derived outcome data-fields with information on date of the participant's baseline recruitment (Date of attending assessment centre) to enable outcomes to be further classified into 'prevalent' (for cases that occurred before recruitment) and 'incident' (for cases that occurred after recruitment). According to current definitions, self-reported events can only be 'prevalent', since information from the repeat assessment nurse-led interviews (performed after recruitment) are not included in the algorithms. The algorithms identify the earliest health-related event of any particular type. To analyse recurrent events, researchers are advised to download all health-related information and develop their own algorithm. (NB. some of the codes in the code list used in this classification might not be indicated for use in analysis of recurrent events). Please be aware that the total number of people with a health-related outcome with source 'Hospital primary' or 'Hospital secondary' does not reflect the total number of participants who have had a health-related hospital admission. Similarly, the total number of people with a health-related outcome with source 'Death primary' or 'Death contributory' does not reflect the total number of participants who have died of that condition; rather, it is the number of people who died with the code(s) on their death record, who did not also have either a hospital admission or self-report event with the relevant code(s). For analysis stratified by source or for summary statistics by source, researchers are advised to download all health-related information and use the codes suggested in the algorithms for the condition(s) of interest.

6. Key points to note

- Great effort has been made to provide the optimal algorithm for the majority of potential research studies. However, different research studies might benefit from using alternative algorithms.
- Estimates of disease frequency in the UK Biobank cohort are not representative of the general (British) population.
- The algorithms and associated data-fields will be updated as additional linked data (especially linked primary care data) are incorporated into the UK Biobank dataset, and with updated information the classification of an individual might change.
- Derived data-fields for other health conditions will be added to the resource as new algorithms are generated.
- Different national data sources were used to classify the health status. Each source provides information for a different range of dates.

Helleryd E, *et al. Open Heart* 2024; 10:e002307. doi: 10.1136/openhrt-2023-002307

7. Health outcome code lists

a) Asthma

UK Biobank Self Report Codes			
**Code Type**	**Code**	**Biobank Code Text**	**Asthma**
UK Biobank Self Report	Field 20002 Code 1111	Asthma	✓
**ICD 9 Codes**			
**Code Type**	**ICD 9 Code**	**ICD 9 Text**	**Asthma**
ICD 9 Code	493	Asthma	✓
ICD 9 Code	493.0	Extrinsic asthma	✓
ICD 9 Code	493.1	Intrinsic asthma	✓
ICD 9 Code	493.19	Intrinsic asthma (without mention of status asthmaticus)	✓
ICD 9 Code	493.2	Chronic obstructive asthma	✓
ICD 9 Code	493.8	Other forms of asthma	✓
ICD 9 Code	493.9	Asthma, unspecified	✓
ICD 9 Code	493.99	Asthma, unspecified (without mention of status asthmaticus)	✓
**ICD 10 Codes**			
**Code Type**	**ICD 10 Code**	**ICD 10 Text**	**Asthma**
ICD 10 Code	J45	Asthma	✓
ICD 10 Code	J45.0	Predominantly allergic asthma	✓
ICD 10 Code	J45.1	Nonallergic asthma	✓
ICD 10 Code	J45.8	Mixed asthma	✓
ICD 10 Code	J45.9	Asthma, unspecified	✓
ICD 10 Code	J46.X[2]	Status asthmaticus	✓

***Acknowledgments**: Code list generated by Qiuli Zhang, Kathryn Bush, John Nolan, Christian Schnier and Cathie Sudlow on behalf of UK Biobank Outcome Adjudication Group.*

[2] An ICD code suffixed with 'X' stands for any code starting with the figures preceding the X

b) Chronic obstructive pulmonary disease (COPD)

UK Biobank Self Report Codes			
**Code Type**	**Code**	**Biobank Code Text**	**COPD**
UK Biobank Self Report	Field 20002 Code 1112	Chronic obstructive airways disease/COPD	✓
UK Biobank Self Report	1113	Emphysema/chronic bronchitis	✓
UK Biobank Self Report	1472	Emphysema	✓
**ICD 9 Codes**			
**Code Type**	**ICD 9 Code**	**ICD 9 Text**	**COPD**
ICD 9 Code	492	Emphysema	✓
ICD 9 Code	492.0	Emphysematous bleb	✓
ICD 9 Code	492.8	Other emphysema	✓
ICD 9 Code	492.9	Emphysema, unspecified	✓
ICD 9 Code	496.X[3]	Chronic airway obstruction, not elsewhere classified	✓
**ICD 10 Codes**			
**Code Type**	**ICD 10 Code**	**ICD 10 Text**	**COPD**
ICD 10 Code	J43	Emphysema	✓
ICD 10 Code	J43.0	MacLeod syndrome	✓
ICD 10 Code	J43.1	Panlobular emphysema	✓
ICD 10 Code	J43.2	Centrilobular emphysema	✓
ICD 10 Code	J43.8	Other emphysema	✓
ICD 10 Code	J43.9	Emphysema, unspecified	✓
ICD 10 Code	J44	Other chronic obstructive pulmonary disease	✓
ICD 10 Code	J44.0	Chronic obstructive pulmonary disease with acute lower respiratory infection	✓
ICD 10 Code	J44.1	Chronic obstructive pulmonary disease with acute exacerbation, unspecified	✓
ICD 10 Code	J44.8	Other specified chronic obstructive pulmonary disease	✓
ICD 10 Code	J44.9	Chronic obstructive pulmonary disease, unspecified	✓

Acknowledgments: *Code list generated by Qiuli Zhang, Kathryn Bush, John Nolan, Christian Schnier, and Cathie Sudlow.*

[3] An ICD code suffixed with 'X' stands for any code starting with the figures preceding the X

c) Dementia

There are no disease-specific ICD codes for Lewy Body Dementia (DLB). The codes commonly used are non-specific and were therefore not included in this algorithm. The codes used include ICD 9 code 331 (Other cerebral degenerations) and ICD 10 code G31.8 (Other specified degenerative diseases of the nervous system). There are however specific codes for DLB included in primary care data (Read codes), which will be included in later versions of this documentation.

During the UKB assessment visit, participants were asked if they had a history of 'Dementia or Alzheimer's or Cognitive Impairment' – to which they answered "Yes" or "No". This code is therefore not specific for dementia or Alzheimer's disease, but has been included in the list of 'All Cause Dementia' for completeness.

A systematic review from the UK Biobank Outcomes Adjudication Group examined the accuracy of identifying dementia cases using routinely collected healthcare data. They reported that, for all-cause dementia, positive predictive values (PPVs) ranged from 33-100%. Sensitivities (relative to all true dementia cases in the population) ranged from 21-86%. PPVs for Alzheimer's disease (range 57-100%) were generally higher than for vascular dementia (range 19-91%).[1]

A validation study in 17,000 Biobank participants in Scotland reported an overall PPV of about 67% for identifying participants with dementia (see Figure 1 below).[2]

Helleryd E, *et al. Open Heart* 2024; 10:e002307. doi: 10.1136/openhrt-2023-002307

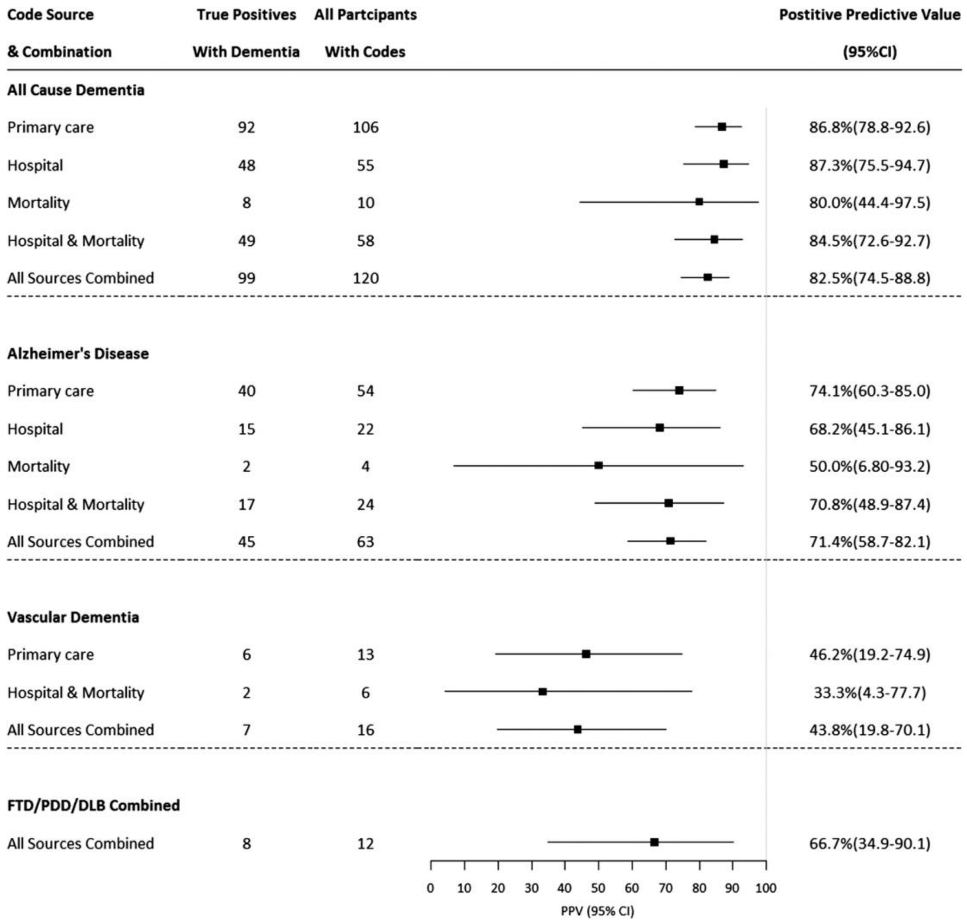

Figure 1. Positive predictive values for datasets, alone and in combination, stratified by dementia subtype. FTD frontotemporal dementia, PDD Parkinson's disease dementia, DLB dementia with Lewy bodies.

References

1. Wilkinson T, Ly A, Schnier C, et al on behalf of the UK Biobank Neurodegenerative Outcomes Group and Dementias Platform UK. Identifying dementia cases with routinely collected health data: A systematic review. Alzheimers Dement. 2018, 14(8):1038-1051.
2. Wilkinson, T., Schnier, C., Bush, K. et al. Identifying dementia outcomes in UK Biobank: a validation study of primary care, hospital admissions and mortality data. Eur J Epidemiol, 2019,34:557–565.

Dementia code lists

UK Biobank Self Report Codes						
**Code Type**	**Code**	**Biobank Code Text**	**AD**	**VD**	**FTD**	**Dementia**
UK Biobank Self Report	Field 20002 Code 1263	Dementia/Alzheimers/Cognitive Impairment				✓
**ICD 9 Codes**						
**Code Type**	**ICD 9 Code**	**ICD 9 Text**	**AD**	**VD**	**FTD**	**Dementia**
ICD 9 Code	290.2	Senile dementia, depressed or paranoid type				✓
ICD 9 Code	290.3	Senile dementia with acute confusional state				✓
ICD 9 Code	290.4	Arteriosclerotic dementia		✓		✓
ICD 9 Code	291.2	Other alcoholic dementia				✓
ICD 9 Code	294.1	Dementia in other conditions classified elsewhere				✓
ICD 9 Code	331.0	Alzheimer's disease	✓			✓
ICD 9 Code	331.1	Pick's disease			✓	✓
ICD 9 Code	331.2	Senile degeneration of brain				✓
ICD 9 Code	331.5	Creutzfeldt-Jakob disease				✓
**ICD 10 Codes**						
**Code Type**	**ICD 10 Code**	**ICD 10 Text**	**AD**	**VD**	**FTD**	**Dementia**
ICD 10 Code	A81.0	Sporadic Creutzfeldt-Jakob disease				✓
ICD 10 Code	F00	Dementia in Alzheimer's disease	✓			✓
ICD 10 Code	F00.0	Dementia in Alzheimer's disease with early onset	✓			✓
ICD 10 Code	F00.1	Dementia in Alzheimer's disease with late onset	✓			✓
ICD 10 Code	F00.2	Dementia in Alzheimer's disease, atypical or mixed type	✓			✓
ICD 10 Code	F00.9	Dementia in Alzheimer's disease, unspecified	✓			✓
ICD 10 Code	F01	Vascular dementia		✓		✓
ICD 10 Code	F01.0	Vascular dementia of acute onset		✓		✓
ICD 10 Code	F01.1	Multi-infarct dementia		✓		✓
ICD 10 Code	F01.2	Subcortical vascular dementia		✓		✓
ICD 10 Code	F01.3	Mixed cortical and sub-cortical vascular dementia		✓		✓

Helleryd E, *et al. Open Heart* 2024; 10:e002307. doi: 10.1136/openhrt-2023-002307

ICD 10 Code	F01.8	Other vascular dementia		✓		✓
ICD 10 Code	F01.9	Vascular dementia, unspecified		✓		✓
ICD 10 Code	F02	Dementia in other diseases classified elsewhere				✓
ICD 10 Code	F02.0	Dementia in Picks disease			✓	✓
ICD 10 Code	F02.1	Dementia in Creutzfeldt-Jacob disease				✓
ICD 10 Code	F02.2	Dementia in Huntington’s disease				✓
ICD 10 Code	F02.3	Dementia in Parkinson’s disease				✓
ICD 10 Code	F02.4	Dementia in HIV disease				✓
ICD 10 Code	F02.8	Dementia in other specified diseases classified elsewhere				✓
ICD 10 Code	F03	Unspecified dementia				✓
ICD 10 Code	F05.1	Delirium superimposed on dementia				✓
ICD 10 Code	F10.6	Mental and behavioural disorders due to use of alcohol - amnesic syndrome				✓
ICD 10 Code	G30	Alzheimer’s disease	✓			✓
ICD 10 Code	G30.0	Alzheimer’s disease with early onset	✓			✓
ICD 10 Code	G30.1	Alzheimer’s disease with late onset	✓			✓
ICD 10 Code	G30.8	Other Alzheimer's disease	✓			✓
ICD 10 Code	G30.9	Alzheimer's disease unspecified	✓			✓
ICD 10 Code	G31.0	Circumscribed brain atrophy			✓	✓
ICD 10 Code	G31.1	Senile degeneration of brain				✓
ICD 10 Code	G31.8	Other specified degenerative diseases of nervous system				✓
ICD 10 Code	I67.3	Binswanger’s disease		✓		

Acknowledgments: *Code list generated by Kathryn Bush, Tim Wilkinson, Christian Schnier, John Nolan and Cathie Sudlow on behalf of UK Biobank Outcome Adjudication Group.*

d) Myocardial infarction (MI)

The estimated accuracy of algorithmically-defined MI events using routine linked health records is based on a systematic review[1] of published studies conducted by the UK Biobank Cardiac Outcomes Group.

The selected ICD codes from hospital data are estimated to produce positive predictive values (PPVs):

- for any MI of 75-100%;
- for STEMI of 71-100%
- for NSTEMI of >90%

The PPV of MI events identified only from death registry data is likely to be somewhat lower (around 70-75%) than those identified in hospital records.

The PPV of MI events diagnosed prior to recruitment and identified by self-report alone is uncertain but likely to be lower than events confirmed in EHR.

Further direct validation studies in UK Biobank participants are ongoing and additional information on accuracy of event identification will be added to this documentation as it becomes available.

References

1. Rubbo, B., Fitzpatrick, N.K., Denaxas, S., et al. Use of electronic health records to ascertain, validate and phenotype acute myocardial infarction: A systematic review and recommendations. Int J Cardiology. 2015, 187:705-711.

MI code lists

UK Biobank Self Report Codes					
**Code Type**	**Field Code 20002**	**Biobank Code Text**	**STEMI**	**NSTEMI**	**MI**
UK Biobank Self Report	1075	Heart attack/myocardial infarction			✓
**ICD 9 Codes**					
**Code Type**	**ICD 9 Code**	**ICD 9 Text**	**STEMI**	**NSTEMI**	**MI**
ICD 9 Code	410	Acute myocardial infarction	✓		✓
ICD 9 Code	410.0	Acute myocardial infarction of anterolateral wall	✓		✓
ICD 9 Code	410.1	Acute myocardial infarction of other anterior wall	✓		✓
ICD 9 Code	410.2	Acute myocardial infarction of inferolateral wall	✓		✓
ICD 9 Code	410.3	Acute myocardial infarction of inferoposterior wall	✓		✓
ICD 9 Code	410.4	Acute myocardial infarction of other inferior wall	✓		✓
ICD 9 Code	410.5	Acute myocardial infarction of other lateral wall	✓		✓
ICD 9 Code	410.6	True posterior wall infarction	✓		✓
ICD 9 Code	410.7	Subendocardial infarction		✓	✓
ICD 9 Code	410.8	Acute myocardial infarction of other specified sites	✓		✓
ICD 9 Code	410.9	Acute myocardial infarction of unspecified site	✓		✓
ICD 9 Code	411.0	Postmyocardial infarction syndrome			✓
ICD 9 Code	412.X[4]	Old myocardial infarction			✓
ICD 9 Code	429.79	Ill-defined descriptions and complications of heart disease – Other			✓
**ICD 10 Codes**					
**Code Type**	**ICD 10 Code**	**ICD 10 Text**	**STEMI**	**NSTEMI**	**MI**
ICD 10 Code	I21	Acute myocardial infarction			✓
ICD 10 Code	I21.0	Acute transmural myocardial infarction of anterior wall	✓		✓
ICD 10 Code	I21.1	Acute transmural myocardial infarction of inferior wall	✓		✓

[4] An ICD code suffixed with 'X' stands for any code starting with the figures preceding the X

ICD 10 Code	I21.2	Acute transmural myocardial infarction of other sites	✓		✓
ICD 10 Code	I21.3	Acute transmural myocardial infarction of unspecified site	✓		✓
ICD 10 Code	I21.4	Acute subendocardial myocardial infarction		✓	✓
ICD 10 Code	I21.9	Acute myocardial infarction, unspecified		✓	✓
ICD 10 Code	I22	Subsequent myocardial infarction			✓
ICD 10 Code	I22.0	Subsequent myocardial infarction of anterior wall	✓		✓
ICD 10 Code	I22.1	Subsequent myocardial infarction of inferior wall	✓		✓
ICD 10 Code	I22.8	Subsequent myocardial infarction of other sites	✓		✓
ICD 10 Code	I22.9	Subsequent myocardial infarction of unspecified site		✓	✓
ICD 10 Code	I23	Certain current complications following acute myocardial infarction			✓
ICD 10 Code	I23.0	Haemopericardium as current complication following acute myocardial infarction			✓
ICD 10 Code	I23.1	Atrial septal defect as current complication following acute myocardial infarction			✓
ICD 10 Code	I23.2	Ventricular septal defect as current complication following acute myocardial infarction			✓
ICD 10 Code	I23.3	Rupture of cardiac wall without haemopericardium as current complication following acute myocardial infarction			✓
ICD 10 Code	I23.4	Rupture of chordae tendineae as current complication following acute myocardial infarction			✓
ICD 10 Code	I23.5	Rupture of papillary muscle as current complication following acute myocardial infarction			✓
ICD 10 Code	I23.6	Thrombosis of atrium, auricular appendage, and ventricle as current complications following acute myocardial infarction			✓
ICD 10 Code	I23.8	Other current complications following acute myocardial infarction			✓
ICD 10 Code	I24.1	Dressler syndrome			✓
ICD 10 Code	I25.2	Old myocardial infarction			✓

Acknowledgments: *Code list generated by Christian Schnier, Kathryn Bush, John Nolan, and Cathie Sudlow on behalf of UK Biobank Outcome Adjudication Group.*

e) Motor neurone disease (MND)

Although ICD 9 and ICD 10 codes exist for MND subtypes, in the UK only the parent codes for MND are used in routine clinical practice. This means that MND subtypes cannot be currently identified from the algorithm. The incorporation of primary care data (Read codes) contains data on sub-types and the algorithm and associated documentation will be updated when these data are available for the full cohort.

The estimated accuracy of algorithmically defined MND events using routine linked health records is based on a systematic review of published studies conducted by the UK Biobank Outcome Adjudication Group, which reported PPVs of between 55 and 92%.[1]

References

1. Horrocks S, Wilkinson T, Schnier C, et al. Accuracy of routinely-collected healthcare data for identifying motor neurone disease cases: A systematic review. Le W, ed. PLoS ONE. 2017;12(2):e0172639. doi:10.1371/journal.pone.0172639

MND code lists

UK Biobank Self Report Codes			
**Code Type**	**Code**	**Biobank Code Text**	**MND**
UK Biobank Self Report	Field 20002 Code 1259	Motor Neurone Disease	✓
**ICD 9 Codes**			
**Code Type**	**ICD 9 Code**	**ICD 9 Text**	**MND**
ICD 9 Code	335.2	Motor Neurone Disease	✓
**ICD 10 Codes**			
**Code Type**	**ICD 10 Code**	**ICD 10 Text**	**MND**
ICD 10 Code	G12.2	Motor Neurone Disease	✓

Acknowledgments: *Code list generated by Kathryn Bush, John Nolan and Cathie Sudlow on behalf of UK Biobank Outcome Adjudication Group.*

f) Parkinson's disease

Some of the clinical features of Parkinson's disease (PD) can be caused by a range of other conditions including Multiple System Atrophy (MSA), Progressive Supranuclear Palsy (PSP), Corticobasal Degeneration (CBD), drug induced Parkinsonism, and others. Therefore, researchers wishing to study PD may wish to consider these other groups in tandem, as they have clinical similarities. It is worth noting that due to the significant clinical overlap between these groups, it is not unusual for people with one of the rarer conditions such as MSA or PSP, to initially receive a diagnosis of PD.[1] In view of this, when using the algorithm to identify participants with PD, researchers may wish to exclude participants with a code for PD, who later also receive a code for MSA, PSP or CBD.

There are no disease specific ICD codes for CBD. The codes commonly used are non-specific and were therefore not included in this algorithm: ICD 9 codes 331 (Other cerebral degenerations) and ICD 10 code: G31.8 (Other specified degenerative diseases of the nervous system). The same codes are also used for Lewy Body Dementia and a range of other conditions. There are however specific codes for CBD from primary care data (Read codes), which will be included in later versions of this documentation.

A validation study of 20,000 Biobank participants in Scotland assessed the accuracy of codes in UK Biobank for identifying participants with PD. In total 78 participants were identified with codes for PD. It was not possible to comment upon the accuracy of identifying participants with MSA/PSP/CBD, due to the small number identified. Figure 2 shows the PPV of codes according to source, which when combined gave a PPV of 91%.

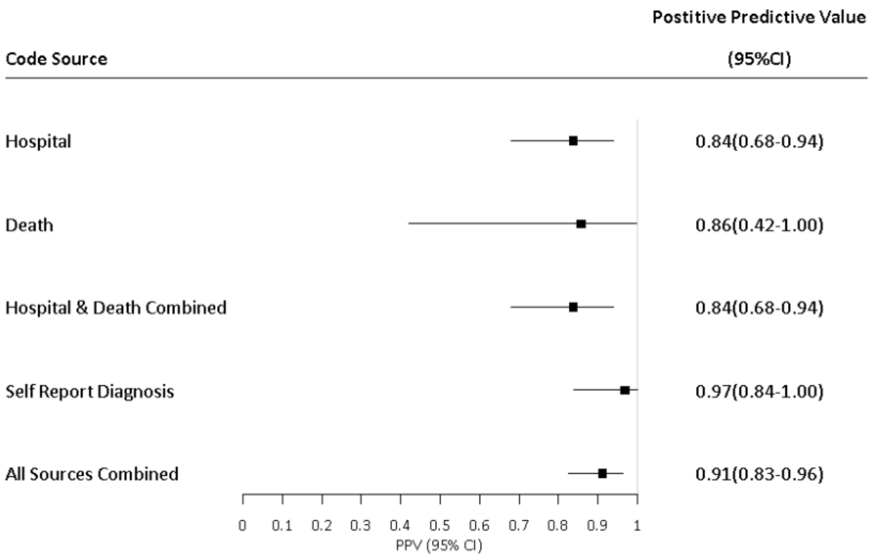

Figure 2. Positive predictive values for codes used to identify PD in UK Biobank.

References

1. Hughes AJ, Daniel SE, Ben-Shlomo Y, et al. The accuracy of diagnosis of parkinsonian syndromes in a specialized movement disorder service. Brain 2002;125:861–70.

Parkinson’s disease code lists

UK Biobank Self Report Codes						
**Code Type**	**Code**	**Biobank Code Text**	**PD**	**MSA**	**PSP**	**All cause Parkinsonism**
UK Biobank Self Report	Field 20002 Code 1262	Parkinson’s disease	✓			✓
**ICD 9 Codes**						
**Code Type**	**ICD 9 Code**	**ICD 9 Text**	**PD**	**MSA**	**PSP**	**All cause Parkinsonism**
ICD 9 Code	3320	Paralysis agitans	✓			✓
ICD 9 Code	3321	Secondary parkinsonism				✓
ICD 9 Code	3330	Other degenerative diseases of basal ganglia				✓
**ICD 10 Codes**						
**Code Type**	**ICD 10 Code**	**ICD 10 Text**	**PD**	**MSA**	**PSP**	**All cause Parkinsonism**
ICD 10 Code	G20	Parkinson’s disease	✓			✓
ICD 10 Code	G21	Secondary parkinsonism				✓
ICD 10 Code	G21.0	Malignant neuroleptic syndrome				✓
ICD 10 Code	G21.1	Other drug induced secondary parkinsonism				✓
ICD 10 Code	G21.2	Secondary parkinsonism due to other external agents				✓
ICD 10 Code	G21.3	Post encephalitic parkinsonism				✓
ICD 10 Code	G21.4	Vascular parkinsonism				✓
ICD 10 Code	G21.8	Other secondary parkinsonism				✓
ICD 10 Code	G21.9	Secondary parkinsonism unspecified				✓
ICD 10 Code	G22	Parkinsonism in diseases specified elsewhere				✓
ICD 10 Code	G23.0	Hallervorden-Spatz disease				✓
ICD 10 Code	G23.1	Progressive Supranuclear Palsy			✓	✓
ICD 10 Code	G23.2	Multiple system atrophy, parkinsonian type [MSA-P]		✓		✓
ICD 10 Code	G23.3	Multiple system atrophy, cerebellar type [MSA-C]		✓		✓
ICD 10 Code	G23.8	Other specified degenerative diseases of basal ganglia (Calcification of basal ganglia Neurogenic orthostatic hypotension [ShyDrager]				✓
ICD 10 Code	G23.9	Degenerative diseases of basal ganglia, unspecified				✓

ICD 10 Code	G25.9	Extrapyramidal and movement disorder, unspecified				✓
ICD 10 Code	G26	Extrapyramidal and movement disorders in diseases classified elsewhere				✓
ICD 10 Code	G90.3	Multi-system degeneration		✓		✓

Acknowledgments: *Code list generated by Kathryn Bush, Kristiina Rannikmae, Tim Wilkinson, Christian Schnier and Cathie Sudlow on behalf of UK Biobank Outcome Adjudication Group.*

f) Stroke

The estimated accuracy of algorithmically defined stroke events is based on two systematic reviews of published studies on coded[1] and self-reported[2] data conducted on behalf of the UK Biobank Stroke Outcomes Group.

The selected ICD codes from hospital and death data are estimated to produce positive predictive values (PPVs):

- for any stroke of around 68-90%;
- for ischaemic stroke of around 66-95%
- for intracerebral haemorrhage of around 71-96%
- for subarachnoid haemorrhage of around 86-96%

The PPV of stroke events prior to recruitment identified by self-report alone is likely to be lower and more variable (22-87%), increasing to 75% or more if transient ischaemic attacks are considered true positives. The PPV of self-report for specific pathological types of stroke is uncertain.

Further validation studies in UK Biobank participants are ongoing and additional information on accuracy of event identification will be added to this documentation as it becomes available.

References

1. Woodfield, R., Grant, I., UK Biobank Stroke Outcomes Group; UK Biobank Follow-Up and Outcomes Working Group, Sudlow, C.L. Accuracy of Electronic Health Record Data for Identifying Stroke Cases in Large-Scale Epidemiological Studies: A Systematic Review from the UK Biobank Stroke Outcomes Group. PLoS One. 2015, 10(10):e0140533.
2. Woodfield, R., UK Biobank Stroke Outcomes Group, UK Biobank Follow-up and Outcomes Working Group, Sudlow, C.L.M. Accuracy of Patient Self-Report of Stroke: A Systematic Review from the UK Biobank Stroke Outcomes Group. PLoS One. 2015, 10(9): e0137538.

Stroke code lists

UK Biobank Self Report Codes						
**Code Type**	**Code**	**Biobank Code Text**	**IS**	**IH**	**SH**	**Stroke**
UK Biobank Self Report	Field 20002 Code 1081	Stroke				✓
UK Biobank Self Report	Field 20002 Code 1086	Subarachnoid haemorrhage			✓	✓
UK Biobank Self Report	Field 20002 Code 1491	Brain haemorrhage		✓		✓
UK Biobank Self Report	Field 20002 Code 1583	Ischaemic stroke	✓			✓
**ICD 9 Codes**						
**Code Type**	**ICD 9 Code**	**ICD 9 Text**	**IS**	**IH**	**SH**	**Stroke**
ICD 9 Code	430.X	Subarachnoid haemorrhage			✓	✓
ICD 9 Code	431.X	Intracerebral haemorrhage		✓		✓
ICD 9 Code	434.X	Occlusion of cerebral arteries	✓			✓
ICD 9 Code	434.0	Cerebral thrombosis	✓			✓
ICD 9 Code	434.1	Cerebral embolism	✓			✓
ICD 9 Code	434.9	Cerebral artery occlusion, unspecified	✓			✓
ICD 9 Code	436.X[5,6]	Acute, but ill-defined, cerebrovascular disease	✓			✓
**ICD 10 Codes**						
**Code Type**	**ICD 10 Code**	**ICD 10 Text**	**IS**	**IH**	**SH**	**Stroke**
ICD 10 Code	I60	Subarachnoid haemorrhage			✓	✓
ICD 10 Code	I60.0	Subarachnoid haemorrhage from carotid siphon and bifurcation			✓	✓
ICD 10 Code	I60.1	Subarachnoid haemorrhage from middle cerebral artery			✓	✓

[5] ICD 10: I64 (Stroke not specified as haemorrhage or infarction) and ICD 9 436 (Acute, but ill-defined, cerebrovascular disease) have been classified as ischaemic stroke because of evidence that the vast majority of these are ischaemic strokes.
[6] An ICD code suffixed with 'X' stands for any code starting with the figures preceding the X

Helleryd E, et al. Open Heart 2024; 10:e002307. doi: 10.1136/openhrt-2023-002307

ICD 10 Code	I60.2	Subarachnoid haemorrhage from anterior communicating artery			✓	✓
ICD 10 Code	I60.3	Subarachnoid haemorrhage from posterior communicating artery			✓	✓
ICD 10 Code	I60.4	Subarachnoid haemorrhage from basilar artery			✓	✓
ICD 10 Code	I60.5	Subarachnoid haemorrhage from vertebral artery			✓	✓
ICD 10 Code	I60.6	Subarachnoid haemorrhage from other intracranial arteries			✓	✓
ICD 10 Code	I60.7	Subarachnoid haemorrhage from intracranial artery, unspecified			✓	✓
ICD 10 Code	I60.8	Other subarachnoid haemorrhage			✓	✓
ICD 10 Code	I60.9	Subarachnoid haemorrhage, unspecified			✓	✓
ICD 10 Code	I61	Intracerebral haemorrhage		✓		✓
ICD 10 Code	I61.0	Intracerebral haemorrhage in hemisphere, subcortical		✓		✓
ICD 10 Code	I61.1	Intracerebral haemorrhage in hemisphere, cortical		✓		✓
ICD 10 Code	I61.2	Intracerebral haemorrhage in hemisphere, unspecified		✓		✓
ICD 10 Code	I61.3	Intracerebral haemorrhage in brain stem		✓		✓
ICD 10 Code	I61.4	Intracerebral haemorrhage in cerebellum		✓		✓
ICD 10 Code	I61.5	Intracerebral haemorrhage, intraventricular		✓		✓
ICD 10 Code	I61.6	Intracerebral haemorrhage, multiple localized		✓		✓
ICD 10 Code	I61.8	Other intracerebral haemorrhage		✓		✓
ICD 10 Code	I61.9	Intracerebral haemorrhage, unspecified		✓		✓
ICD 10 Code	I63	Cerebral infarction	✓			✓
ICD 10 Code	I63.0	Cerebral infarction due to thrombosis of precerebral arteries	✓			✓
ICD 10 Code	I63.1	Cerebral infarction due to embolism of precerebral arteries	✓			✓

ICD 10 Code	I63.2	Cerebral infarction due to unspecified occlusion or stenosis of precerebral arteries	✓			✓
ICD 10 Code	I63.3	Cerebral infarction due to thrombosis of cerebral arteries	✓			✓
ICD 10 Code	I63.4	Cerebral infarction due to embolism of cerebral arteries	✓			✓
ICD 10 Code	I63.5	Cerebral infarction due to unspecified occlusion or stenosis of cerebral arteries	✓			✓
ICD 10 Code	I63.6	Cerebral infarction due to cerebral venous thrombosis, nonpyogenic	✓			✓
ICD 10 Code	I63.8	Other cerebral infarction	✓			✓
ICD 10 Code	I63.9	Cerebral infarction, unspecified	✓			✓
ICD 10 Code	I64.X[4]	Stroke, not specified as haemorrhage or infarction	✓			✓

Acknowledgments: *Code list generated by Christian Schnier, Kathryn Bush, John Nolan and Cathie Sudlow on behalf of UK Biobank Outcome Adjudication Group.*

Acknowledgements

We would like to acknowledge the contributions of the 'UK Biobank Follow-up and Outcomes Working Group', whose work provided the foundations of the original documentation on UK Biobank's ADOs:

Chair: John Danesh, Cambridge University
Naomi Allen, UK Biobank, Oxford University
Mark Atkinson, Swansea University
Ekaterini Blaveri, Cancer Research UK
Rachael Brannan, National Cancer Intelligence Network
Carol Brayne, Cambridge University
Sinead Brophy, Swansea University
Nish Chaturvedi, University College London
Rory Collins, UK Biobank, Oxford University
Simon deLusignan, Surrey University
Spiros Denaxas, University College London
Parul Desai, Moorfields Eye Hospital
Sophie Eastwood, University College London
John Gallacher, Cardiff University
Harry Hemingway, University College London
Matthew Hotopf, Kings College London
Martin Landray, Oxford University
Ronan Lyons, Swansea University
Mark McGilchrist, Dundee University
Henrik Moller, Kings College London
Terence O'Neil, Manchester University
Mike Pringle, Nottingham University
Tim Sprosen, Oxford University
David Strachan, St George's University, London
Cathie Sudlow, UK Biobank, Edinburgh University
Frank Sullivan, Dundee University
Rebecca Woodfield, Edinburgh University
Qiuli Zhang, UK Biobank, Edinburgh University
Secretariat: Robin Flaig, UK Biobank Edinburgh University
