# Supplementary material for: Association between exercise load, resting heart rate, and maximum heart rate and risk of future ST-segment elevation myocardial infarction (STEMI)
Source: Open Heart. 2023 Jul 17;10(2):e002307. doi: 10.1136/openhrt-2023-002307 (PMC10357634; doi:10.1136/openhrt-2023-002307)
Supplement: Supplementary data [file openhrt-2023-002307supp002.pdf]

Supplementary Table 1 - Cox proportional hazard model with LDL and without diastolic heart rate and weight

	coef	exp(coef)	se(coef)	z	p
**Resting heart rate**	0.0122775	1.0123531	0.0056107	2.188	0.028654
**Age**	0.0365338	1.0372094	0.0108114	3.379	0.000727
**Male**	1.7944328	6.0160613	0.2036352	8.812	< 2e-16
**Systolic Blood pressure**	0.0054229	1.0054376	0.0037311	1.453	0.146109
**Pack year of smoking**	0.0141625	1.0142633	0.0033448	4.234	2.29e-05
**BMI**	-0.0337518	0.9668114	0.0176711	-1.910	0.056133
**FVC**	-0.4303360	0.6502906	0.2199839	-1.956	0.050440
**FEV1**	0.0526497	1.0540603	0.2975236	0.177	0.859540
**PEF**	0.0007406	1.0007409	0.0007662	0.967	0.333744
**Diabetes**	0.7706934	2.1612643	0.2401100	3.210	0.001329
**LDL**	0.4652768	1.5924549	0.0782650	5.945	2.77e-09

Supplementary Table 2 - Cox proportional hazard model with use of beta blockers

	coef	exp(coef)	se(coef)	z	p
**Resting Heart rate**	0.0140646	1.0141640	0.0055834	2.519	0.011769
**Age**	0.0358517	1.0365021	0.0105229	3.407	0.000657
**Male**	1.7393933	5.6938881	0.2138628	8.133	4.18e-16
**Systolic Blood Pressure**	0.0059438	1.0059615	0.0046711	1.272	0.203206
**Diastolic Blood Pressure**	0.0062040	1.0062233	0.0087836	0.706	0.479995
**Pack year of smoking**	0.0121315	1.0122053	0.0032689	3.711	0.000206
**BMI**	0.0037389	1.0037459	0.0384752	0.097	0.922586
**Weight**	-0.0118498	0.9882201	0.0117883	-1.005	0.314793
**FVC**	-0.3861091	0.6796964	0.2199349	-1.756	0.079163
**FEV1**	0.1098163	1.1160730	0.2869396	0.383	0.701931
**PEF**	0.0006818	1.0006821	0.0007286	0.936	0.349350
**Diabetes**	0.3532034	1.4236206	0.2219907	1.591	0.111593
**Beta Blockers**	0.1642614	1.1785224	0.2474453	0.664	0.506800
